# Strategies for Prevention and Management of Hypertension throughout Life

**DOI:** 10.2188/jea.14.112

**Published:** 2005-03-18

**Authors:** Katsuyuki Miura

**Affiliations:** 1 Department of Epidemiology and Public Health, Kanazawa Medical University, Ishikawa, Japan

**Keywords:** blood pressure, hypertension, prevention, epidemiology

## Abstract

Hypertension has been acknowledged as one of the greatest and established risk factors for cardiovascular diseases. In this special article, strategies for the prevention and management of hypertension throughout human’s life were discussed. Studies showing the relationship of birth weight and height increase in childhood to future blood pressure suggest that both environments during pregnancy and during childhood and adolescence are important to prevent hypertension. The promotion of a DASH (Dietary Approach to Stop Hypertension) dietary pattern, rich in fruits and vegetables, is important not only for treatment of high blood pressure but also for long-term prevention of blood pressure rise as well. Blood pressure measured in young adulthood can effectively predict long-term risks of cardiovascular and all-cause mortality, so population-wide primary prevention of high blood pressure for young adults is important. Recent large scale cohort studies confirmed that detection and evaluation of hypertension based mainly on systolic blood pressure remains the most practical and easy approach in the general population for young adult, middle-aged, and older men and women. Researchers in Asia are desired to establish high-quality epidemiologic evidences for Asian for the prevention and management of hypertension.

## Introduction

Hypertension has been acknowledged as one of the greatest and established risk factors for cardiovascular diseases (heart diseases and stroke). Particularly in Asian countries, where the mortality and morbidity of stroke are higher than in western countries, measures against hypertension are considered very important in the prevention of stroke.^1^^-^^3^ Hypertension affects a majority of the elderly, and drug therapies for hypertension have greatly added to medical costs in most developed countries. The conquest of hypertension is now a major challenge.^4^

This special article is written on the occasion that I was given the Young Investigator’s Award of the Japan Epidemiological Association in January 2004. I have been involved mainly in epidemiologic researches on the prevention and management of hypertension, and the award was given for these researches. In this article, I would attempt to discuss strategies for the prevention and management of hypertension throughout human’s life, showing results from my related research papers.

## Birth weight, childhood growth, and future blood pressure

Recent epidemiologic studies have demonstrated that birth weight and other measures of prenatal growth are associated with adult blood pressure (BP)^5^^-^^7^ and with cardiovascular disease mortality in later life.^8^^-^^11^ On the other hand, it has also been suggested in Western populations that short stature is an important risk factor for cardiovascular diseases.^12^^-^^16^ Although Barker et al hypothesized that malnutrition during pregnancy is responsible for the development of short stature and overweight as well as of several risk factors in adult life, including hypertension,^17^ socioeconomic conditions that persist throughout life can cause both lower birth weight and slower increase in height during adolescence.^18^ Some cross-sectional within-population studies, especially in children and adolescents, have shown a positive relationship between height and BP,^19^^-^^21^ and another inter-population study showed inverse relation between height and BP.^16^ Thus, the underlying mechanism of the association of height with cardiovascular disease is not yet clear. However, findings on the effect of height increase during childhood, independent of birth weight, on cardiovascular disease and its major risk factors are limited.

To determine whether birth weight and childhood growth, especially height increase rate, independently relate to BP in adult life, we conducted a 20-year follow-up study, using the record-linkage method, in a Japanese population.^22^ In this study, both birth weight and rate of height increase in childhood and adolescence were inversely and independently associated with BP at age 20 years (Table 1). These results suggest that both environments during pregnancy and during childhood and adolescence independently affect subsequent BP level.

**Table 1.** Predicted differences in systolic blood pressure (mmHg) at age 20 for 1 standard deviation higher values of birth weight, % increase in height, and weight at age 20 estimated by multiple linear regression analysis, among 2,198 men and 2,428 women born in 1965-1974, Ishikawa, Japan.

	Men			Women		
	
	1 standard deviation	Predicted difference	95% confidence interval	1 standard deviation	Predicted difference	95% confidence interval
Model 1						
Birth weight	0.44 kg	-1.5	-2.0 , -1.0	0.41 kg	-1.0	-1.4 , -0.5
Weight at age 20	9.4 kg	4.4	4.0 , 4.9	7.3 kg	3.3	2.9 , 3.7
Model 2						
Birth weight	0.44 kg	-1.6	-2.1 , -1.1	0.41 kg	-1.0	-1.4 , -0.5
% increase in height (age 3 to 20)	5.4 %	-0.7	-1.1 , -0.2	4.9 %	-0.5	-0.9 , -0.1
Weight at age 20	9.4 kg	4.5	4.0 , 5.0	7.3 kg	3.4	3.0 , 3.8

Both models are also adjusted for gestational age.

Modified from reference 22.

## Food intake and long-term BP increase

By the early 1990’s, based on scientific evidence available at the time, nutritional guidelines for prevention and control of high blood pressure recommended weight control, reduced intake of sodium chloride (salt), avoidance of heavy alcohol consumption, and increased dietary potassium intake.^23^^,^^24^ The DASH (Dietary Approach to Stop Hypertension) study recently added further dietary approaches to reduce BP in both nonhypertensive and hypertensive individuals using a “combination” dietary pattern during an 8-week intervention.^25^^-^^27^ This dietary pattern emphasizes higher than usual intakes of fruit and vegetables and low-fat dairy products. It also includes selection of whole grains, poultry, fish, and nuts, and reduced intake of total fats, saturated fats, cholesterol, red meats, sweets, and sugar-containing beverages. This diet is high in potassium, magnesium, phosphorus, calcium, fiber, and protein. The DASH-Sodium trial also showed that the combination diet plus reduced salt intake (at about 50 mmol/day) yielded substantial combined reductions in BP of both nonhypertensive and hypertensive persons. The DASH dietary concept, emphasizing a healthy pattern based on food rather than nutrient intake, has been incorporated into recent dietary guidelines by the American Heart Association^28^ and by JNC7 (the Seventh Report of the Joint National Committee on Prevention, Detection, Evaluation, and Treatment of High Blood Pressure).^29^ While the DASH combination diet is effective in lowering blood pressure, influences of specific foods and food groups (e.g., vegetables, fruits, fish, red meats) on BP have not been well studied long-term. Information is especially sparse on relationships of food groups to BP change in populations followed prospectively for years.

We reported relationships of food intake to BP change in a prospective cohort study of 1,710 middle-aged men, the Chicago Western Electric Study.^30^ The Generalized Estimating Equation method was used to analyze relationships of food group intakes to average annual BP change, adjusting for age, weight at each year, alcohol, calories, and other foods. Men who consumed 0.5-1.5 cups vegetables/day were estimated to have 2.8 mmHg less systolic BP (SBP) rise in 7 years than men who consumed <0.5 cups/day. Men who consumed 0.5-1.5 cups fruit/day were estimated to have 2.2 mmHg less SBP increase in 7 years than men who consumed <0.5 cups/day. Beef-veal-lamb intake and poultry intake were related directly to greater BP rise. These findings lend further support to the promotion of a DASH-style dietary pattern not only for treatment of high blood pressure but also for long-term prevention of BP rise as well.

## BP in young adults can predict future cardiovascular mortality

For middle-aged and older populations worldwide, BP has repeatedly been shown to be a significant risk factor for coronary heart disease (CHD), stroke, and the major cardiovascular diseases (CVD).^1^^-^^3^ These relationships, for both SBP and diastolic BP (DBP), are continuous, graded, strong, independent of other risk factors, consistent, predictive, and generally assessed as etiologically significant. In contrast, long-term observations on blood pressure and CHD-CVD mortality in young adults are limited. Because major CVD events are rare before age 50 years in men and 60 in women, studies on risk factors measured at an average age of about 30 years require long-term follow-up or large sample sizes to accrue adequate numbers of events. The few reports of prospective population-based studies are from nested case-control investigations in former college students.^31^^-^^33^ Other evidence comes from autopsy studies showing that coronary risk factors relate to early atherosclerotic lesions in young adults.^34^^-^^36^ Although hypertension treatment guidelines are usually considered applicable for persons ages 18 and older,^23^^,^^24^^,^^29^ there is limited documentation supporting screening and treatment of young adults.

**Figure 1.**
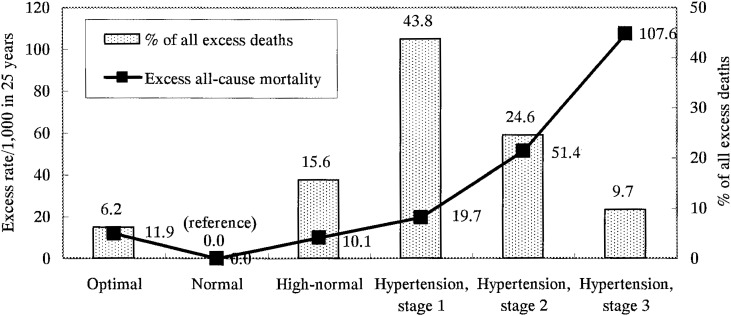
Absolute excess risk per 1,000 in 25 years and percentage of all excess deaths from all causes in strata of JNC-VI classification^23^ in 10,874 men aged 18-39 years at baseline, the Chicago Heart Association Detection Project in Industry. Percentage of all excess deaths was calculated from estimated number of excess deaths compared with the normal blood pressure stratum by JNC-VI criteria during 25 years of follow-up. Modified from reference 37.

The Chicago Heart Association Detection Project in Industry Study is one of the largest and longest prospective studies providing CVD mortality data. Approximately 11,000 men ages 18-39 at baseline (30 years on average) were followed for 25 years, and we reported the relationship of baseline BP to 25-year CHD, CVD, and all-cause mortality.^37^ The main findings on this cohort of young adult men are: (1) BP measured in young adulthood predicted long-term risks of CHD, CVD, and all-cause mortality. As in middle-aged and older persons,^1^^-^^3^ relationships of SBP, DBP, and SBP/DBP (JNC-VI strata^23^) to mortality were generally graded, strong, and independent. (2) Multivariate-adjusted HRs tended to be greater for SBP than DBP, and similar in size to those for middle-aged men. And (3) for the two large strata with high-normal BP and with stage 1 hypertension, 25-year absolute risks and absolute excess risks of mortality were substantial, e.g., all causes death rates of 63 and 72 per 1,000, absolute excess rates of 10 and 20 per 1,000, translating into estimated shorter life expectancy of 2.2 and 4.1 years. These two strata accounted for 59% of all excess deaths attributable to SBP/DBP above normal (Figure 1).

These data lend strong support to two strategic concepts: First, the importance of population-wide primary prevention by safe nutritional-hygienic means of adverse BP levels, so that a substantial increase is achieved in the proportion of people in the population who, throughout life, have favorable levels of BP (and other risk factors). Second, population-wide efforts for early detection of children, teenagers, and young adults – as well as others – with unfavorable BP levels, so that therapeutic efforts can be instituted early, first and foremost to improve lifestyles.

## Importance of SBP and DBP in various age groups

Some recent epidemiologic studies reported that pulse pressure (PP), the difference between SBP and DBP, is a useful predictor for CHD or total CVD especially in middle-aged or older people.^38^^-^^41^ These reports emphasized the importance of PP as a CHD or CVD risk factor, especially because PP is often higher after age 50, apparently due to increased arterial stiffness.^42^^,^^43^ In regard to prior reports on PP, many did not compare the significance of PP with that of SBP or DBP, and some others were studies on hypertensive persons only. Therefore, it was uncertain whether PP is superior to SBP or DBP in predicting future CHD, CVD, and all-cause deaths in various age-sex groups of the general (i.e., apparently healthy) population. Moreover, because recent discussions have emphasized the importance of SBP compared to DBP,^44^^-^^46^ and these are strongly correlated, it is also important to assess whether DBP has any additional role in predicting risks independently from SBP.

We reported relations of four BP indices — PP, SBP, DBP, and mean arterial pressure (MAP) — to 25-year mortality risks from CHD, CVD, and all causes in five population cohorts (total 28,360 men and women) classified by age and sex from the Chicago Heart Association Detection Project in Industry Study.^47^ The main findings from this study were: (1) Relationships of PP were less strong than those of SBP for all endpoints in all age-sex groups studied. (2) Relationships of PP were less strong than those of DBP for all endpoints in middle-aged men and women and in older women. (3) Among the four BP indices, the strongest relationship was observed either for SBP or MAP in all age-sex groups. (4) Relationships of SBP to mortality tended to be stronger than or similar to those of DBP. And (5) with control for SBP, DBP was positively and significantly related to mortality in middle-age men and women, but not in younger men and older men and women.

As to implications of these results for public health policy and clinical practice: (1) They affirm continued emphasis on SBP,^44^^-^^46^ particularly for younger men and older people. For middle-aged people ages 40-59, DBP should be given concomitant careful consideration because of its strong independent relationship to mortality. (2) In younger and middle-aged people, emphasis on PP should be avoided. There is no evidence, in a general population less than age 60, showing that PP is superior to SBP in predicting CVD or total mortality. Emphasizing risks associated with PP is likely to underestimate true risks. And (3) relationships of MAP to risk were generally as strong as or slightly stronger than those of SBP. However, use of this index may not be practical in daily clinical and public health practice, because there are no guidelines for hypertension diagnosis and management using MAP. Detection and evaluation of hypertension based mainly on SBP remains the most practical and easy approach in the general population for young adult, middle-aged, and older men and women (at least up to about age 63 years). After this report, several cohort studies including older people did the same kind of analysis and showed that the relationship of PP to mortality from total cardiovascular diseases and coronary heart disease was less strong than those of other BP indexes.^48^^-^^50^

## Making evidences for Asian people

Very fortunately, I was able to contribute to long-term, large-scale cohort studies in Chicago. However, most study participants in these studies were Caucasian, and, as it is commonly true for many other major hypotheses in preventive cardiology, epidemiologic evidences for Asian people tend to be sparse. Recently I have had a good opportunity to participate in the INTERnational cooperative study of MAcro- and micro-nutrients and blood Pressure (the INTERMAP) and found that there are big differences in body mass, fat intake, cigarette smoking, etc., between Asian and Western people.^51^^,^^52^ Strategies for the prevention and management of hypertension and cardiovascular diseases for Asian would be different from those for Western people. Researchers in Asia should establish high-quality epidemiologic evidences for Asian and accomplish the prevention of hypertension and cardiovascular diseases throughout life.

## References

[1] Stamler J, Stamler R, Neaton JD. Blood pressure, systolic and diastolic, and cardiovascular risks: US population data. Arch Intern Med 1993; 153: 598-615. 8439223 10.1001/archinte.153.5.598

[2] MacMahon S, Peto R, Cutler J, Collins R, Sorlie P, Neaton J, . Blood pressure, stroke, and coronary heart disease: part 1, prolonged differences in blood pressure: prospective observation studies corrected for the regression dilution bias. Lancet 1990; 335: 765-74. 1969518 10.1016/0140-6736(90)90878-9

[3] Eastern Stroke and Coronary Heart Disease Collaborative Research Group. Blood pressure, cholesterol, and stroke in eastern Asia. Lancet 1998; 352: 1801-7. 9851379

[4] Miura K, Daviglus ML, Greenland P, Stamler J. Making prevention and management of hypertension work. J Hum Hypertens 2001;15:1-3. 11223996 10.1038/sj.jhh.1001142

[5] Law CM, Shiell AW. Is blood pressure inversely related to birth weight? The strength of evidence from a systemic review of the literature. J Hypertens 1996;14:935-41. 8884547

[6] Law CM, de Swiet M, Osmond C, Fayers PM, Barker DJ, Cruddas AM, . Initiation of hypertension in utero and its amplification throughout life. BMJ 1993;306:24-7. 8435572 10.1136/bmj.306.6869.24 PMC1676382

[7] Curhan GC, Willet WC, Rimm EB, Spiegelman D, Ascherio AL, Stampfer MJ. Birth weight and adult hypertension, diabetes mellitus, and obesity in US men. Circulation 1996;94:3246-50. 8989136 10.1161/01.cir.94.12.3246

[8] Barker DJP, Winter PD, Osmond C, Margetts B, Simmonds SJ. Weight in infancy and death from ischaemic heart disease. Lancet 1989;ii:577-80. 10.1016/s0140-6736(89)90710-1 2570282

[9] Martyn CN, Barker DJP, Osmond C. Mothers’ pelvic size, fetal growth, and death from stroke and coronary heart disease in men in the UK. Lancet 1996;348:1264-8. 8909378 10.1016/s0140-6736(96)04257-2

[10] Frankel S, Elwood P, Sweetnam P, Yarnell J, Smith GD. Birthweight, body-mass index in middle age, and incident coronary heart disease. Lancet 1996;348:1478-80. 8942776 10.1016/S0140-6736(96)03482-4

[11] Rich-Edwards JW, Stampfer MJ, Manson JE, Rosner B, Hankinson SE, Colditz GA, . Birth weight and risk of cardiovascular disease in a cohort of women followed up since 1976. BMJ 1997;315:396-400. 9277603 10.1136/bmj.315.7105.396 PMC2127275

[12] Miura K, Nakagawa H, Greenland P. Height-cardiovascular disease relation: where to go from here? Am J Epidemiol 2002;155:688-9. 11943684 10.1093/aje/155.8.688

[13] Marmot MG, Rose G, Shipley M, Hamilton PJ. Employment grade and coronary heart disease in British civil servants. J Epidemiol Comm Health 1978;32:244-9. 10.1136/jech.32.4.244 PMC1060958 744814

[14] Rich-Edwards JW, Manson JE, Stampfer MJ, Colditz GA, Willett WC, Rosner B, . Height and the risk of cardiovascular disease in women. Am J Epidemiol 1995;142:909-17. 7572971 10.1093/oxfordjournals.aje.a117738

[15] Njolstad I, Arnesen E, Lund-Larsen PG. Body height, cardiovascular risk factors, and risk of stroke in middle-aged men and women: A 14-year follow-up of the Finnmark study. Circulation 1996;94:2877-82. 8941116 10.1161/01.cir.94.11.2877

[16] Whincup PH, Cook DG, Adshead F, Taylor S, Papacosta O, Walker M, . Cardiovascular risk factors in British children from towns with widely differing adult cardiovascular mortality. BMJ 1996;313:79-84. 8688758 10.1136/bmj.313.7049.79 PMC2351475

[17] Barker DJP, Gluckman PD, Godfrey KM, Harding JE, Owens JA, Robinson JS. Fetal nutrition and cardiovascular disease in adult life. Lancet 1993;341:938-41. 8096277 10.1016/0140-6736(93)91224-a

[18] Crouse JR 3rd. Reduced height for weight and cardiovascular disease. Lancet 1993;341:931-2. 8096271 10.1016/0140-6736(93)91218-b

[19] Rosner B, Prineas RJ, Loggie JM, Daniels SR. Blood pressure nomograms for children and adolescents, by height, sex, and age, in the United States. J Pediatr 1993;123:871-86. 8229519 10.1016/s0022-3476(05)80382-8

[20] Hashimoto N, Kawasaki T, Kikuchi T, Uchiyama M. Criteria of normal blood pressure and hypertension in Japanese preschool children. J Hum Hypertens 1997;11:351-4. 9249228 10.1038/sj.jhh.1000440

[21] Rona RJ, Qureshi S, Chinn S. Factors related to total cholesterol and blood pressure in British 9 year olds. J Epidemiol Community Health 1996;50:512-8. 8944856 10.1136/jech.50.5.512 PMC1060341

[22] Miura K, Nakagawa H, Tabata M, Morikawa Y, Nishijo M, Kagamimori S. Birth weight, childhood growth, and cardiovascular disease risk factors in Japanese aged 20 years. Am J Epidemiol 2001;153:783-9. 11296151 10.1093/aje/153.8.783

[23] The Joint National Committee of Prevention, Detection, Evaluation, and Treatment of High Blood Pressure. The sixth report of The Joint National Committee of Prevention, Detection, Evaluation, and Treatment of High Blood Pressure. Arch Intern Med 1997;157:2413-46. 9385294 10.1001/archinte.157.21.2413

[24] The Guidelines Subcommittee of the World Health Organization - International Society of Hypertension (WHO- ISH) Mild Hypertension Liaison Committee. 1999 World Health Organization - International Society of Hypertension Guidelines for the Management of Hypertension. J Hypertens 1999;17:151-83. 10067786

[25] Appel LJ, Moore TJ, Obarzanek E, Vollmer WM, Svetkey LP, Sacks FM, . A clinical trial of the effects of dietary patterns on blood pressure. N Engl J Med 1997;336:1117-24. 9099655 10.1056/NEJM199704173361601

[26] Sacks FM, Svetkey LP, Vollmer WM, Appel LJ, Bray GA, Harsha D, . Effects on blood pressure of reduced dietary sodium and the Dietary Approaches to Stop Hypertension (DASH) diet. DASH-Sodium Collaborative Research Group. N Engl J Med 2001;344:3-10. 11136953 10.1056/NEJM200101043440101

[27] Svetkey LP, Simons-Morton D, Vollmer WM, Appel LJ, Conlin PR, Ryan DH, . Effects of dietary patterns on blood pressure: subgroup analysis of the Dietary Approaches to Stop Hypertension (DASH) randomized clinical trial. Arch Intern Med 1999;159:285-93. 9989541 10.1001/archinte.159.3.285

[28] Krauss RM, Eckel RH, Howard B, Appel LJ, Daniels SR, Deckelbaum RJ, . AHA Dietary Guidelines: revision 2000: A statement for healthcare professionals from the Nutrition Committee of the American Heart Association. Circulation 2000;102:2284-99. 11056107 10.1161/01.cir.102.18.2284

[29] Chobanian AV, Bakris GL, Black HR, Cushman WC, Green LA, Izzo JL Jr, . The Seventh Report of the Joint National Committee on Prevention, Detection, Evaluation, and Treatment of High Blood Pressure: the JNC 7 report. JAMA 2003;289: 2560-72. 12748199 10.1001/jama.289.19.2560

[30] Miura K, Greenland P, Stamler J, Liu K, Daviglus ML, Nakagawa H. Relation of vegetable, fruit, and meat intake to 7-year blood pressure change in middle-aged men: the Chicago Western Electric Study. Am J Epidemiol 2004;159:572-80. 15003961 10.1093/aje/kwh085

[31] Paffenbarger RS Jr, Notkin J, Krueger DE, Wolf PA, Thorne MC, LeBauer EJ, . Chronic disease in former college students. II. Methods of study and observations on mortality from coronary heart disease. Am J Public Health 1966; 56: 962-71. 10.2105/ajph.56.6.962 PMC1257109 5949327

[32] Paffenbarger RS Jr, Wing AL. Characteristics in youth predisposing to fatal stroke in later years. Lancet 1967; 1: 753-4. 4164123 10.1016/s0140-6736(67)91367-0

[33] Paffenbarger RS Jr, Wing AL. Chronic disease in former college students. X. The effects of single and multiple characteristics on risk of fatal coronary heart disease. Am J Epidemiol 1969; 90: 527-35. 5362860 10.1093/oxfordjournals.aje.a121099

[34] Berenson GS, Srinivasan SR, Bao W, Newman WP III, Tracy RE, Wattigney WA; for the Bogalusa Heart Study. Association between multiple cardiovascular risk factors and atherosclerosis in children and young adults. N Engl J Med 1998; 338: 1650-6. 9614255 10.1056/NEJM199806043382302

[35] Newman WP III, Freedman DS, Voors AW, Gard PD, Srinivasan SR, Cresanta JL, . Relation of serum lipoprotein levels and systolic blood pressure to early atherosclerosis: the Bogalusa Heart Study. N Engl J Med 1986; 314: 138-44. 3455748 10.1056/NEJM198601163140302

[36] McGill HC Jr, McMahan CA, Tracy RE, Oalmann MC, Cornhill JF, Herderick EE, . Relation of a postmortem renal index of hypertension to atherosclerosis and coronary artery size in young men and women: Pathological Determinants of Atherosclerosis in Youth (PDAY) Research Group. Arterioscler Thromb Vasc Biol 1998; 18: 1108-18. 9672071 10.1161/01.atv.18.7.1108

[37] Miura K, Daviglus ML, Dyer AR, Liu K, Garside DB, Stamler J, . Relationship of blood pressure to 25-year mortality due to coronary heart disease, cardiovascular diseases, and all causes in young adult men: the Chicago Heart Association Detection Project in Industry. Arch Intern Med 2001;161:1501-8. 11427097 10.1001/archinte.161.12.1501

[38] Franklin SS, Khan SA, Wong ND, Larson MG, Levy D. Is pulse pressure useful in predicting risk for coronary heart disease? The Framingham Heart Study. Circulation 1999; 100: 354-60. 10421594 10.1161/01.cir.100.4.354

[39] Benetos A, Rudnichi A, Safar M, Guize L. Pulse pressure and cardiovascular mortality in normotensive and hypertensive subjects. Hypertension 1998;32:560-4. 9740626 10.1161/01.hyp.32.3.560

[40] Benetos A, Safar M, Rudnichi A, Smulyan H, Richard JL, Ducimetieere P, . Pulse pressure: a predictor of long-term cardiovascular mortality in a French male population. Hypertension 1997; 30: 1410-5. 9403561 10.1161/01.hyp.30.6.1410

[41] Madhavan S, Ooi WL, Cohen H, Alderman MH. Relation of pulse pressure and blood pressure reduction to the incidence of myocardial infarction. Hypertension 1994;23:395-401. 8125567 10.1161/01.hyp.23.3.395

[42] Franklin SS, Gustin W IV, Wong ND, Larson MG, Weber MA, Kannel WB, . Hemodynamic patterns of age-related changes in blood pressure: The Framingham Heart Study. Circulation 1997;96:308-15. 9236450 10.1161/01.cir.96.1.308

[43] Safer ME. Pulse pressure in essential hypertension: clinical and therapeutical implications. J Hypertens 1989;7:769-776. 2685115 10.1097/00004872-198910000-00001

[44] Black HR. The paradigm has shifted, to systolic blood pressure. Hypertension 1999;34:386-7. 10489381 10.1161/01.hyp.34.3.386

[45] Lloyd-Jones DM, Evans JC, Larson MG, O’Donnell CJ, Levy D. Differential impact of systolic and diastolic blood pressure level on JNC-VI staging. Hypertension 1999;34:381-5. 10489380 10.1161/01.hyp.34.3.381

[46] Kannel WB. Elevated systolic blood pressure as a cardiovascular risk factor. Am J Cardiol 2000;85:251-255. 10955386 10.1016/s0002-9149(99)00635-9

[47] Miura K, Dyer AR, Greenland P, Daviglus ML, Hill M, Liu K, . Pulse pressure compared with other blood pressure indexes in the prediction of 25-year cardiovascular and all-cause mortality rates: The Chicago Heart Association Detection Project in Industry Study. Hypertension 2001;38:232-7. 11509482 10.1161/01.hyp.38.2.232

[48] Domanski M, Mitchell G, Pfeffer M, Neaton JD, Norman J, Svendsen K, . Pulse pressure and cardiovascular disease-related mortality: follow-up study of the Multiple Risk Factor Intervention Trial (MRFIT). JAMA 2002;287:2677-83. 12020303 10.1001/jama.287.20.2677

[49] Prospective Studies Collaboration. Age-specific relevance of usual blood pressure to vascular mortality: a meta-analysis of individual data for one million adults in 61 prospective studies. Lancet 2002;360:1903-13. 12493255 10.1016/s0140-6736(02)11911-8

[50] Asia Pacific Cohort Studies Collaboration. Blood pressure indices and cardiovascular disease in the Asia Pacific region: a pooled analysis. Hypertension 2003;42:69-75. 12756223 10.1161/01.HYP.0000075083.04415.4B

[51] Stamler J, Elliott P, Dennis B, Dyer AR, Kesteloot H, Liu K, . INTERMAP: background, aims, design, methods, and descriptive statistics (nondietary). J Hum Hypertens 2003;17:591-608. 13679950 10.1038/sj.jhh.1001603 PMC6660162

[52] Zhou BF, Stamler J, Dennis B, Moag-Stahlberg A, Okuda N, Robertson C, . Nutrient intakes of middle-aged men and women in China, Japan, United Kingdom, and United States in the late 1990s: the INTERMAP study. J Hum Hypertens 2003;17:623-30. 13679952 10.1038/sj.jhh.1001605 PMC6561109

